# Supplementary figures and images for: RT-Sort: An action potential propagation-based algorithm for real time spike detection and sorting with millisecond latencies
Source: PLoS One. 2024 Dec 5;19(12):e0312438. doi: 10.1371/journal.pone.0312438 (PMC11620616; doi:10.1371/journal.pone.0312438)

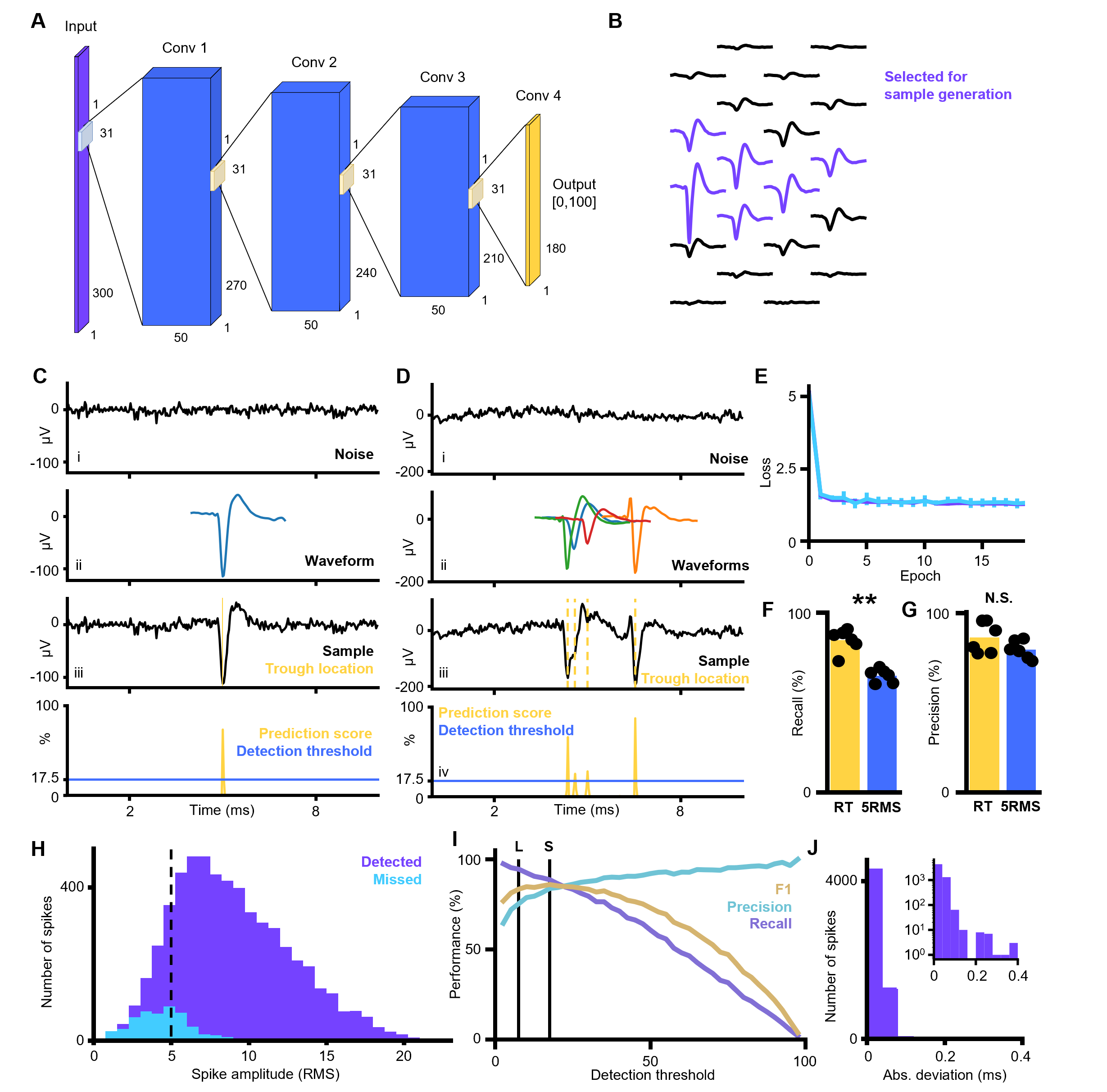

Supplement: S1 Fig — A) Architecture of the CNN used for spike detection in 30kHz Neuropixels recordings. The input layer consists of 300 nodes, corresponding to a 10ms window. The output layer consists of 180 nodes corresponding to 2-8ms in the input window. Each output node provides a score between 0 and 100 indicating the likelihood that the corresponding input frame contains a waveform trough. The 4 convolutional layers have a kernel size of 31 frames and a stride of 1 frame so that each output node makes a prediction based on the signal in 2ms before until 2ms after the corresponding frame in the input. B) Example of averaged waveform footprint detected by Kilosort2. The purple traces were selected for training/validating the CNN. C) Example of training/validating sample creation and model prediction. i: A 10ms sample of recording specific noise is taken. ii: A waveform shape is selected from the training or validating pool. iii: The waveform is pasted into the recording device specific noise with ground truth certainty about the trough location (marked with yellow dotted line). iv: CNN detection model predictions for the frames in 2-8ms of the input window show a narrow detection peak at the waveform trough. All figures share the same x-axis. D) Same as C but with multiple overlapping waveforms in the same sample. E) Training (purple) and validating (cyan) loss as a function of training epoch. Error bars indicate the STD over the different cross-validation folds. The small differences between the training loss and validating loss indicate that the models is not overfitting nor underfitting the data. F) Recall when validating the detection model on samples generated from the held-out recording and when applying a 5RMS threshold to the same samples. The markers indicate the results for each of the 6 held-out datasets and the bar reflects the mean over all held-out datasets. Mean±STD for CNN = 84.7%±5.81% and for 5RMS = 65.1%±3.37%. The detection model has a significantly higher reca [file pone.0312438.s001.tif]

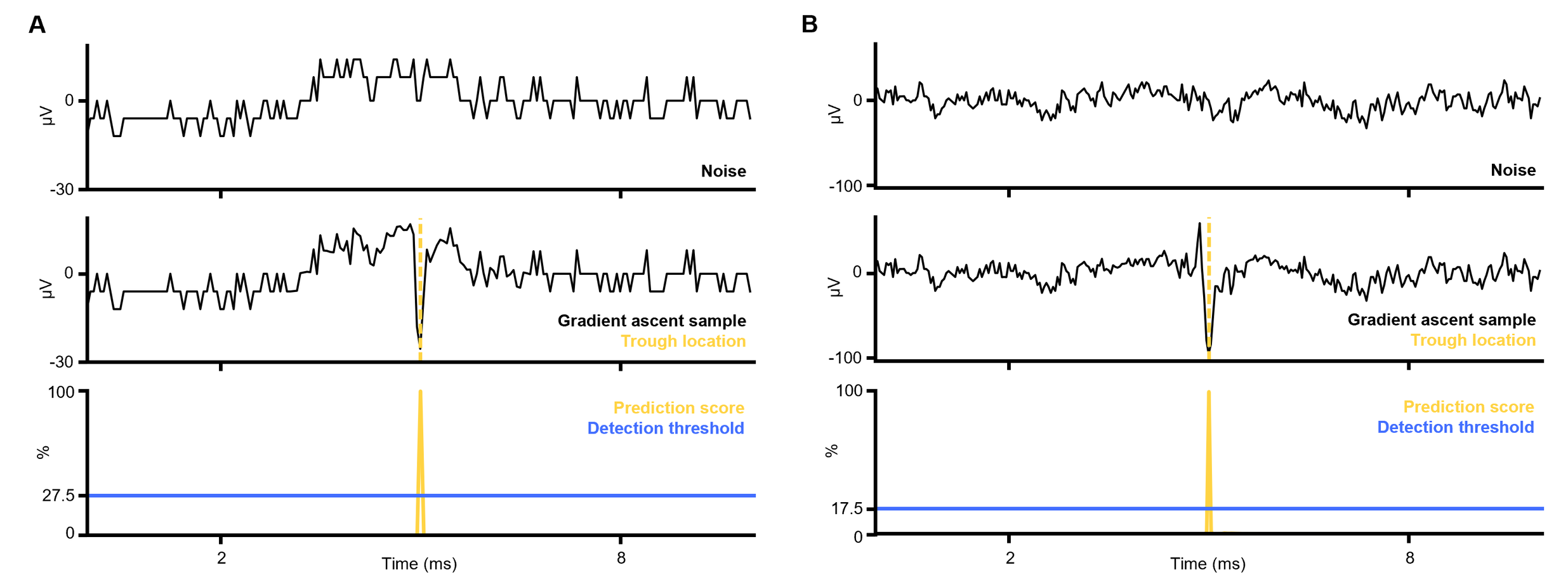

Supplement: S2 Fig — A) (top) Intrinsic noise from an MEA electrode. (middle) Same piece of intrinsic noise after using the MEA spike detection model to perform gradient ascent on the noise to generate a waveform shape that leads to a detection with 100% certainty at the center of the noise sample. (bottom) Prediction scores for the noise sample after completing gradient ascent. B) Same as A but for Neuropixels noise using the Neuropixels spike detection model. (TIF) [file pone.0312438.s002.tif]

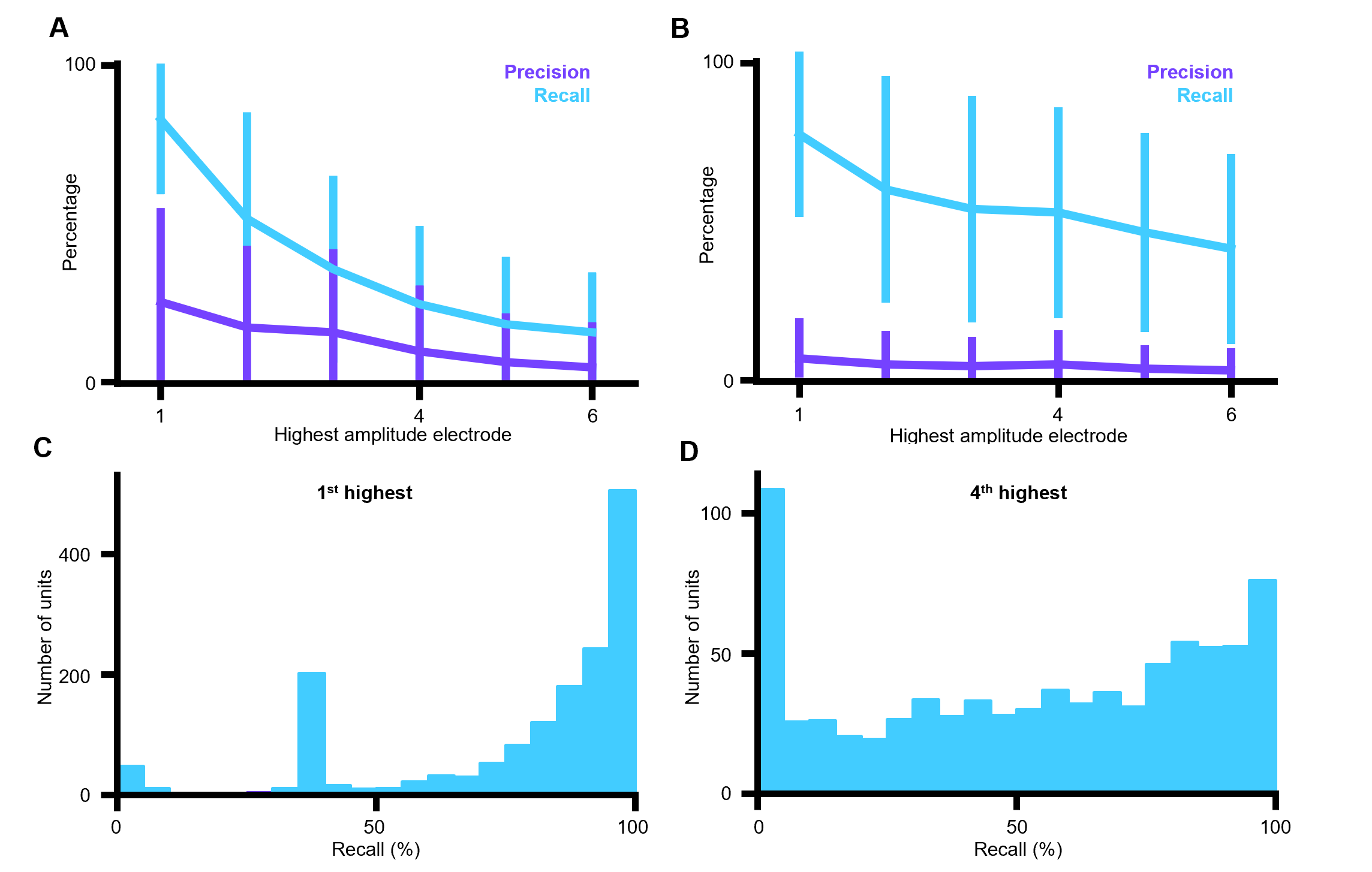

Supplement: S3 Fig — A) Precision and recall of CNN spike detection model using the ith highest amplitude electrode for each Kilosort2 detection as ground truth. The Kilosort2 detections for 6 organoid MEA recordings are grouped together and the spike detection model is used to detect spikes on the highest amplitude electrode (1 on x-axis) down to the 6th highest amplitude electrode (6 on x-axis). The line indicates the mean and the error bars the standard deviation over all units from all different recordings. B) Same as A but for the 6 mouse in vivo Neuropixels recordings. C) Recall distribution over all highest amplitude electrodes from the Neuropixels recordings. D) Recall distribution over all the 4th highest amplitude electrodes from the Neuropixels recordings. (TIF) [file pone.0312438.s003.tif]

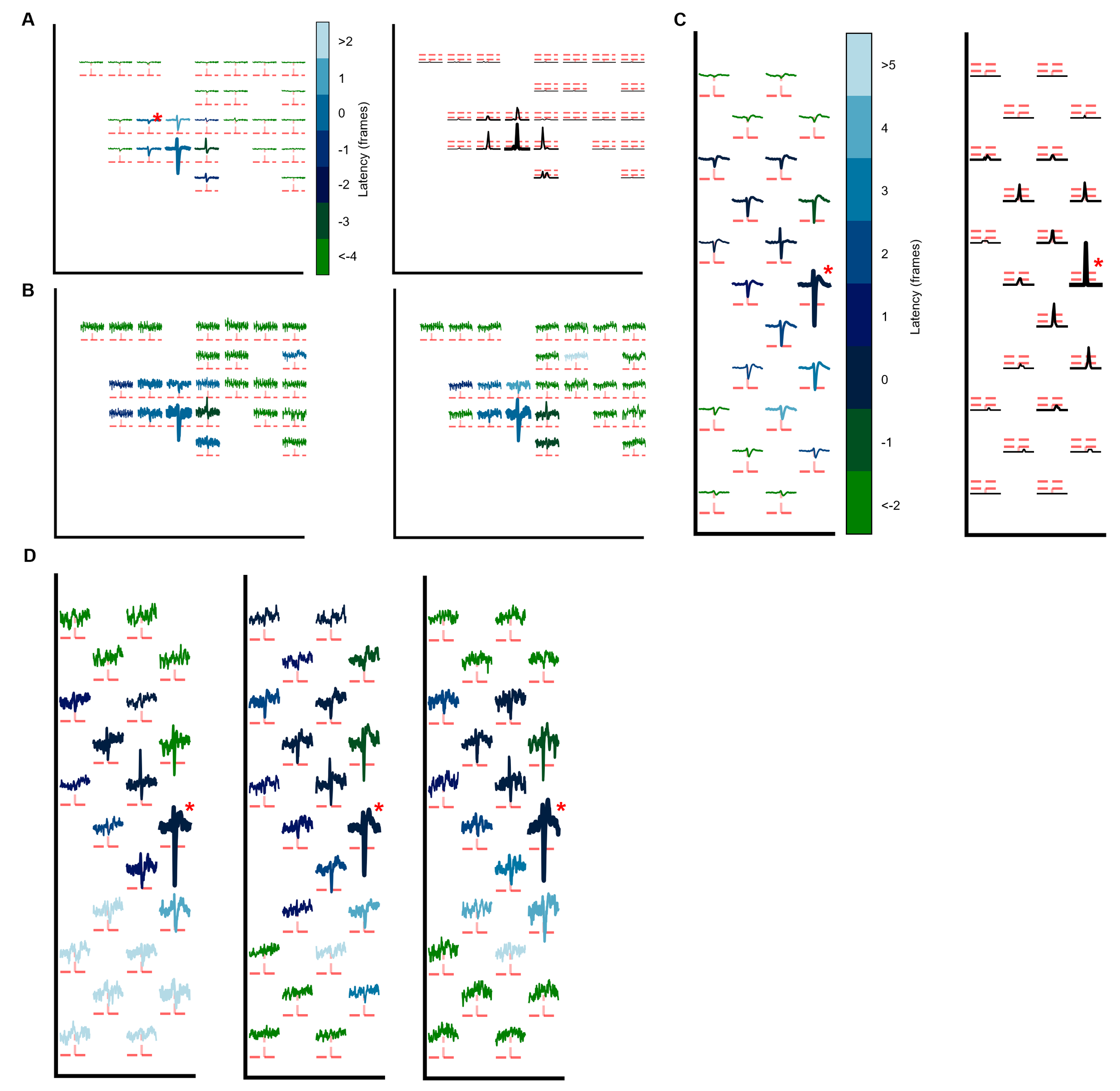

Supplement: S4 Fig — A) (left) Averaged waveform footprint of unit recorded with patch-clamp and MEA and detected by RT-Sort. (right) Corresponding averaged spike detection model footprint. Lines, colors and markers have the same meaning as Fig 2A, 2B. B) Examples of single spikes from the detected unit in A using RT-Sort in online mode. Color scale is the same as in A. Lines, colors and markers have the same meaning as Fig 2D. C) (left) Waveform footprint of unit recorded with Neuropixels probe and detected by RT-Sort. (right) corresponding averaged spike detection footprint over all detected action potentials. Lines, colors and markers have the same meaning as Fig 2A, 2B. D) Examples of single spikes from the detected unit in C. Color scale and markers are the same as in panel C. (TIF) [file pone.0312438.s004.tif]

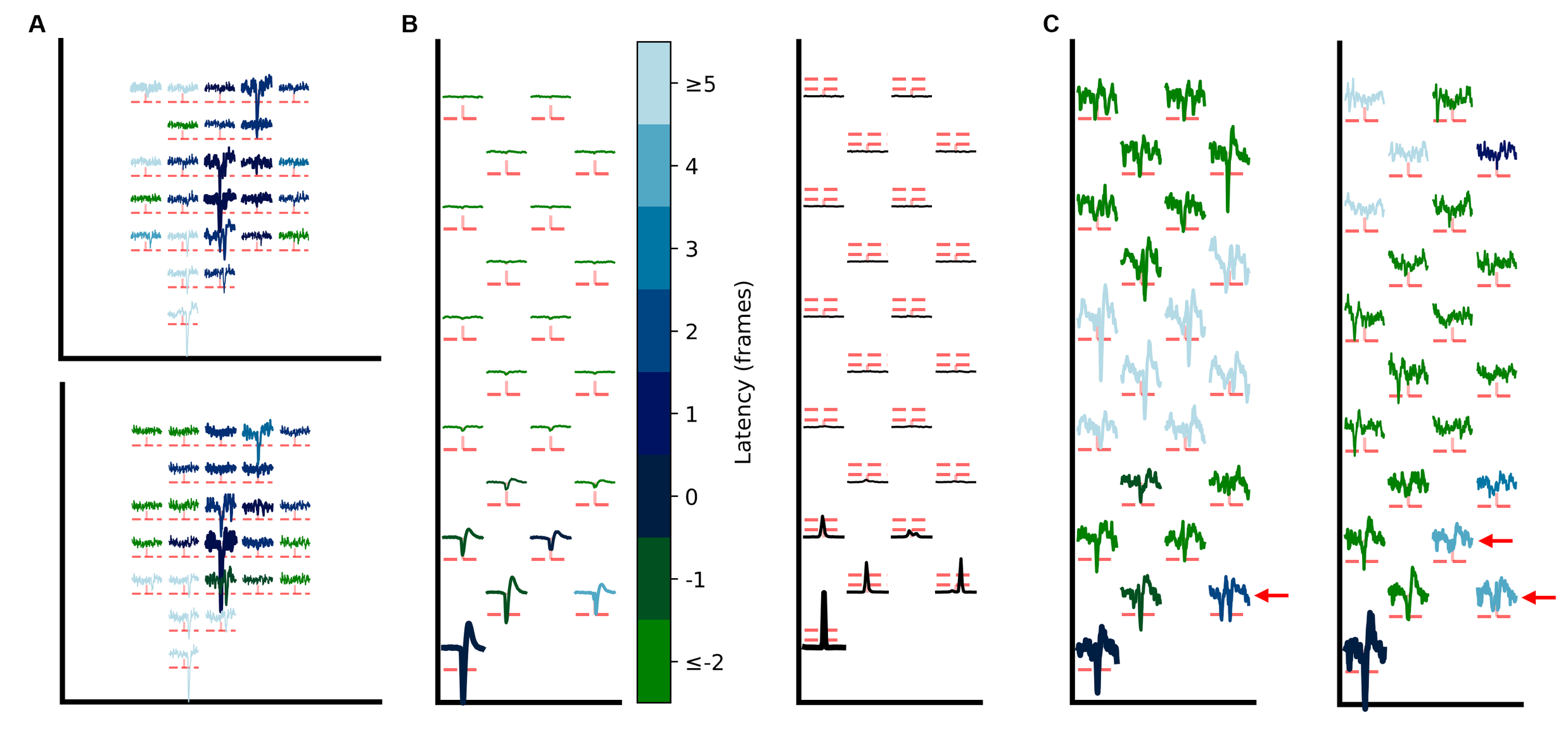

Supplement: S5 Fig — A) Additional examples of overlapping waveform spikes from the unit in Fig 2A that are correctly detected by RT-Sort. Lines, colors and markers have the same meaning as in Fig 2D. B) (left) Averaged waveform footprint of unit recorded with Neuropixels probe and detected by RT-Sort. (right) Corresponding averaged spike detection model footprint. Lines, colors and markers have the same meaning as Fig 2A, 2B. C) Examples of individual overlapping waveform spikes from the unit in panel B. Color scale and markers are the same as in B. (TIF) [file pone.0312438.s005.tif]

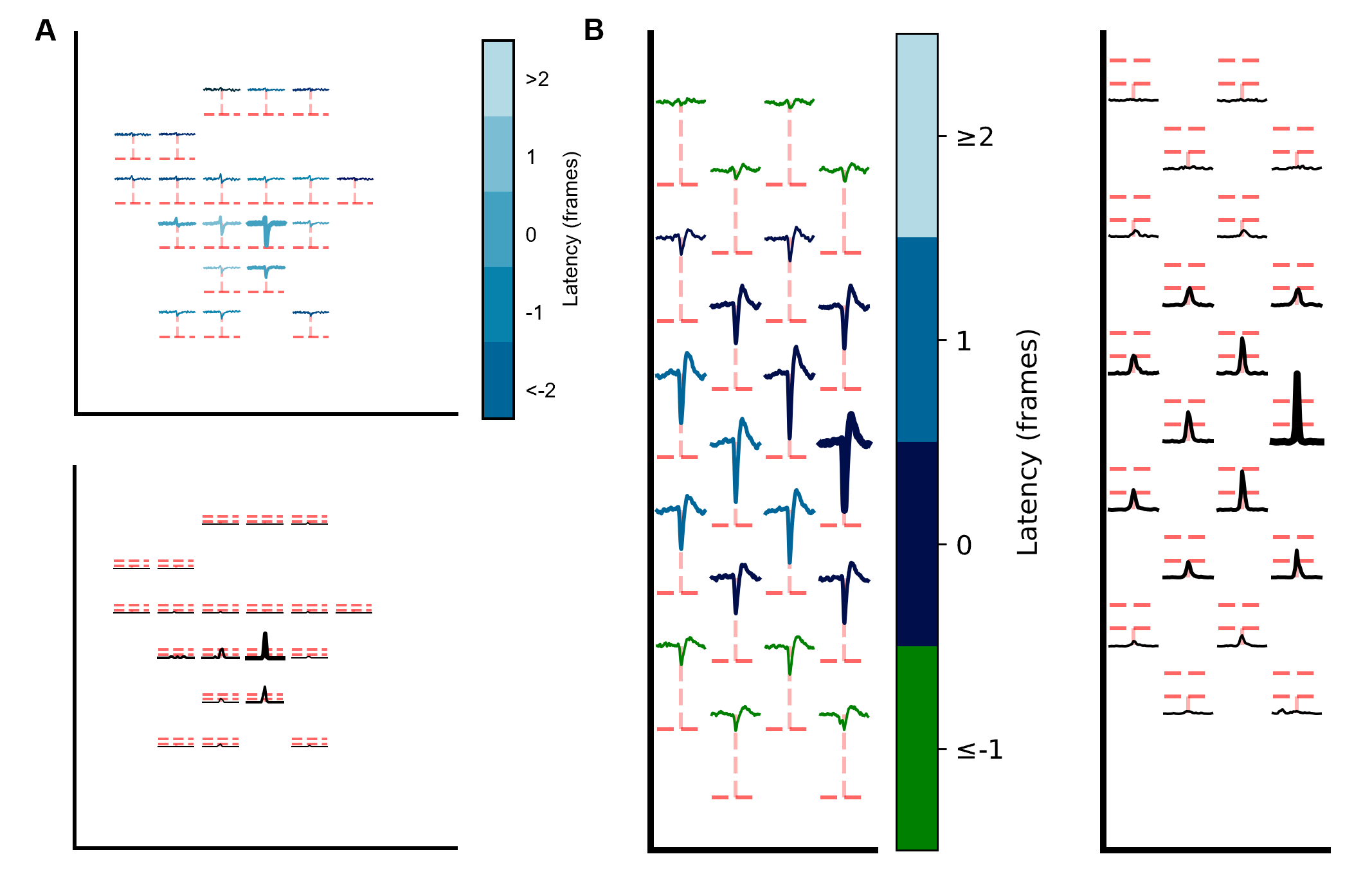

Supplement: S6 Fig — A) (top) Averaged waveform footprint of unit recorded with MEA and detected by RT-Sort. All electrodes record the unit below 5 times the signal to noise ratio, marked per electrode with the dotted red line. (bottom) Corresponding averaged CNN detection footprint. 4 electrodes detect the action potential above the loose detection threshold marked per electrode with the bottom dotted red line. Lines, colors and markers have the same meaning as Fig 2A, 2B. B) (left) Averaged waveform footprint of unit recorded with Neuropixels and detected by RT-Sort. All electrodes record the unit below 5 times the signal to noise ratio, marked per electrode with the dotted red line. (right) Corresponding averaged spike detection model footprint. 10 electrodes detect the action potential above the loose detection threshold marked per electrode with the bottom dotted red line. Lines, colors and markers have the same meaning as Fig 2A, 2B. (TIF) [file pone.0312438.s006.tif]

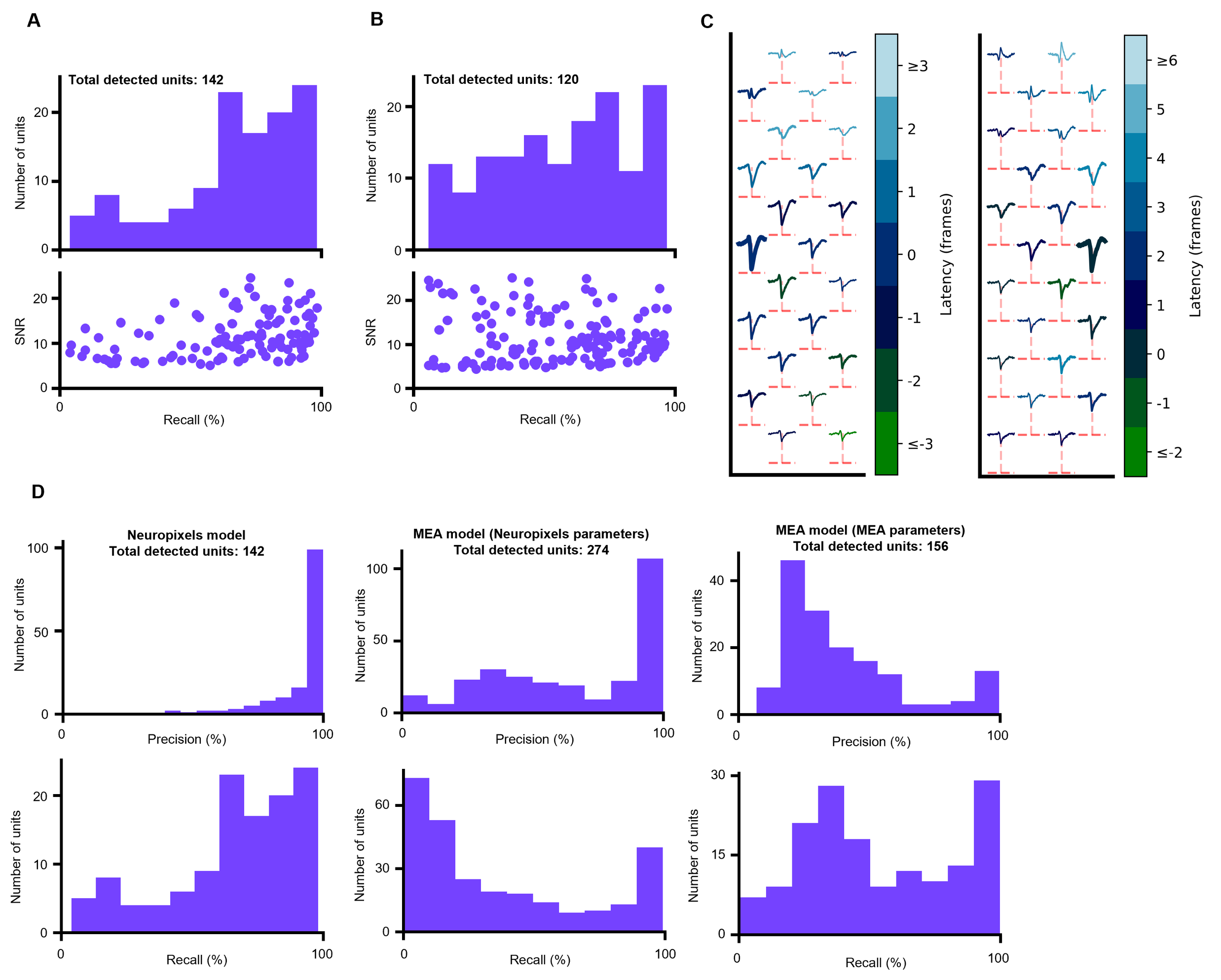

Supplement: S7 Fig — A) Top: for each unit detected in the simulated ground truth recording using sequence metrics generated based on the ground truth spike locations, the recall over all detected spikes compared to the most similar ground truth neuron. Mean±STD = 70.9%±25.0%. Bottom: for each unit shown in the histogram at the top of the panel, the corresponding average waveform amplitude expressed in SNR. Top and bottom share the same x-axis. B) Top: for each unit detected in the simulated ground truth recording, the recall over all detected spikes compared to the most similar ground truth neuron. Mean±STD = 60.4%±25.9%. Bottom: for each unit shown in the histogram at the top of the panel, the corresponding average waveform amplitude expressed in SNR. Top and bottom share the same x-axis. C) Examples of averaged waveform footprints from units from the simulated ground truth recording with unrealistic waveform shapes that did not get detected by RT-Sort (overlap score <0.01). Lines, colors and markers have the same meaning as Fig 2D. D) Precision and recall when applying the MEA spike detection model on the simulated Neuropixels recordings using either the Neuropixels parameters (middle) or the MEA parameters (right). For comparison, the results of applying the Neuropixels model as presented in Fig 2I and S7A Fig are included on the left. (TIF) [file pone.0312438.s007.tif]

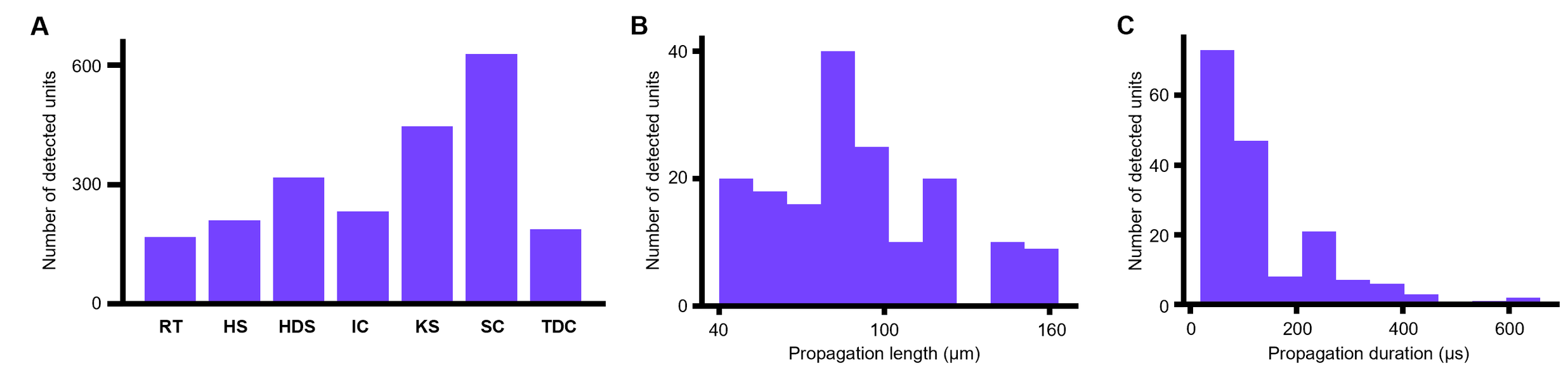

Supplement: S8 Fig — A) Number of units detected by different spike sorting algorithms in the recording for Fig 4. Abbreviations: RT = RT-Sort, HS = Herdingspikes2, HDS = HD-Sort, IC = IronClust, KS = Kilosort2, SC = SpyKing Circus, TDC = Tridesclous. B) Distribution of propagation lengths for all detected RT-Sort units in the same recording as panel A. C) Distribution of all propagation durations for all detected RT-Sort units in the same recording as panel A. (TIF) [file pone.0312438.s008.tif]

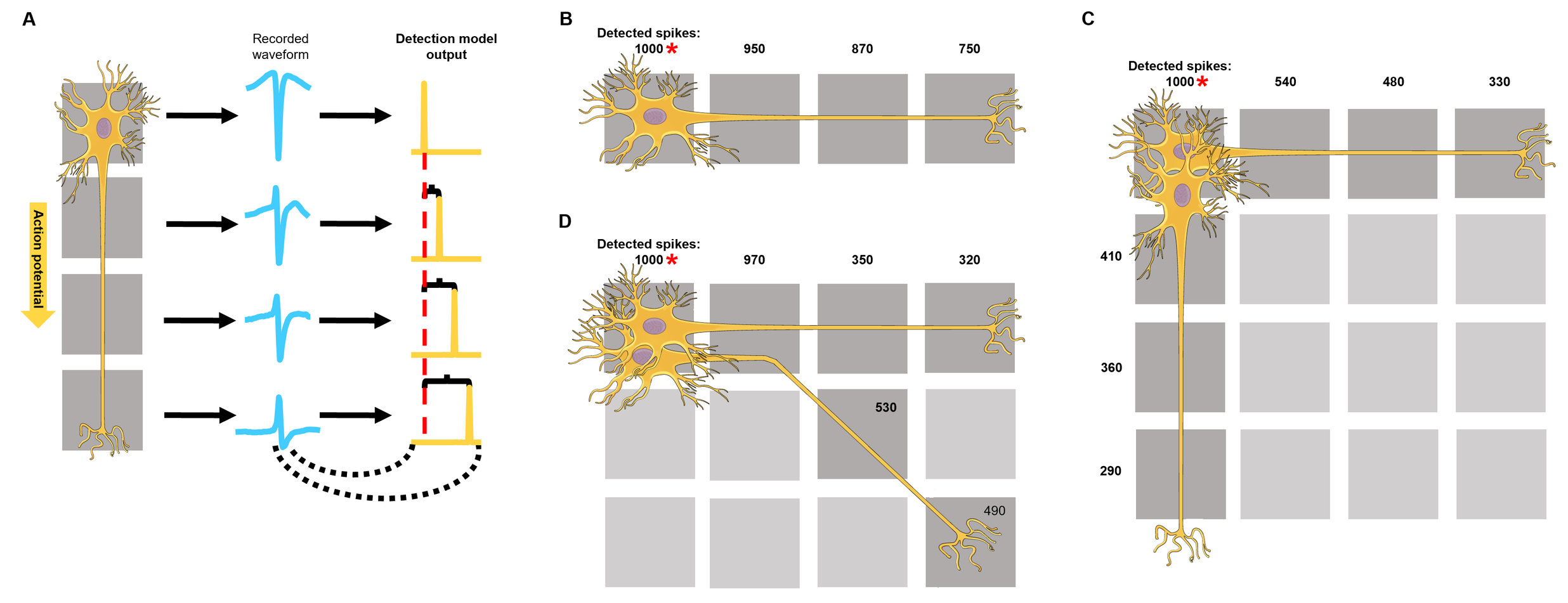

Supplement: S9 Fig — A) An action potential traveling down an axon and recorded by different electrodes (represented as gray squares) can yield varying detected waveform shapes on each electrode. The CNN spike detection model can identify these waveforms as spikes and provides a highly sensitive estimation of the waveform trough detection time on each electrode (the detection model output on the right covers 0.5ms whereas the waveform shapes in the middle cover 5ms). Using these CNN spike detection model outputs, the action potential propagation duration can be measured for the propagating action potential. B) Example of an action potential propagation measured by 4 electrodes. In this example, the root electrode, marked with a red star, detects 1000 spikes. Subsequent electrodes that co-detect a spike within 0.5ms of a spike detected on the root electrode are used as splitting electrodes in order of increasing distance. The nearest electrode is used as the first splitting electrode to assess whether the current subset of root detections are composed of spikes from multiple neurons; in this example, they are not. The subset of root detections that coincide with spikes on the splitting electrode are considered to be the new selection of root detections when the next closest electrode is assessed. C) Similar as B but in this example, two different neurons share the same root electrode. Root detections that are considered to be part of the propagation sequence of one neuron can not also be part of the propagation sequence of the other neuron. D) Similar as C but in this case the neurons diverge only after having shared a first splitting electrode after the root electrode. For the subsequent electrodes, detections that are considered to be part of the propagation sequence of one neuron can not also be part of the propagation sequence of the other neuron. (TIF) [file pone.0312438.s009.tif]

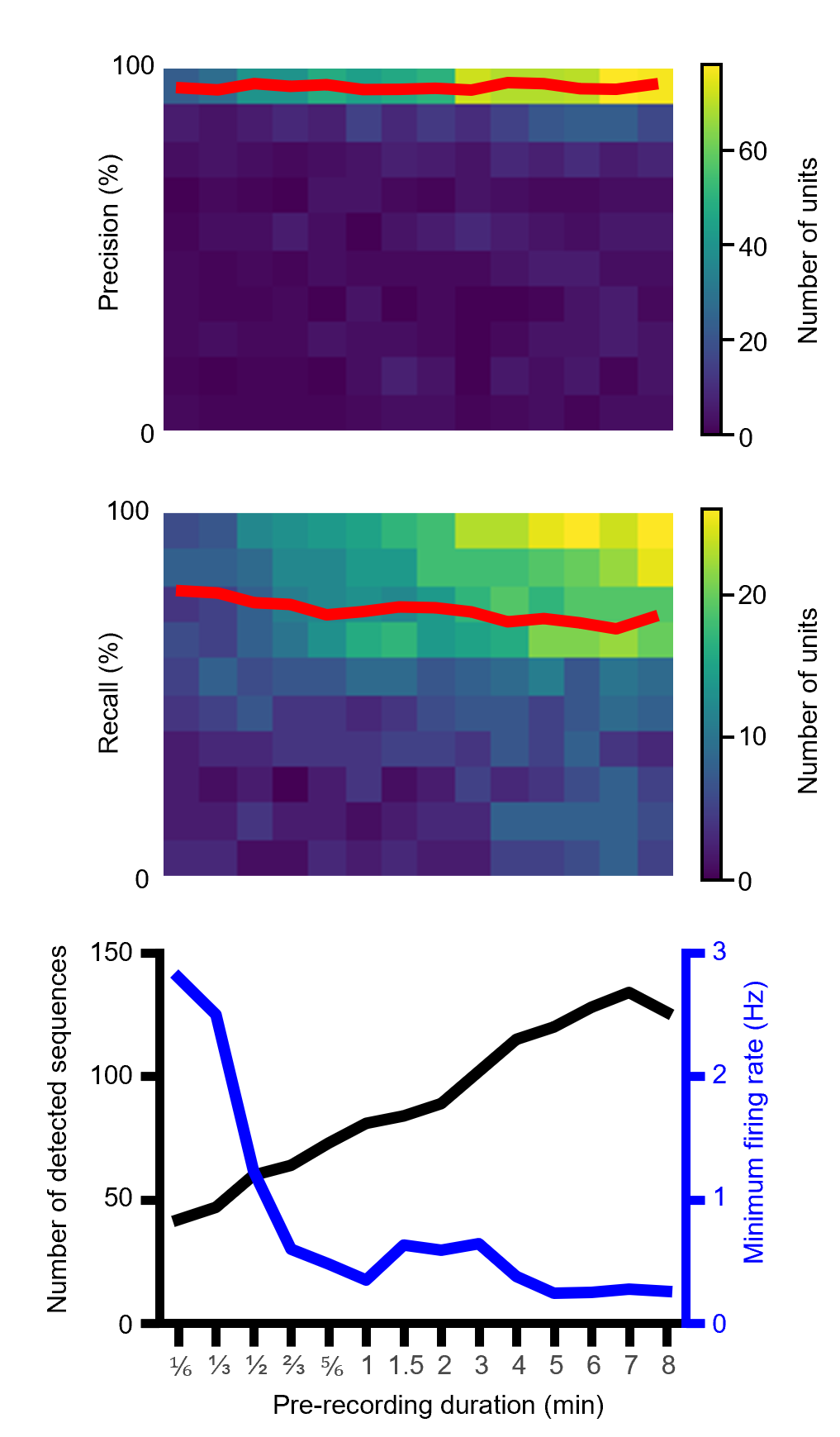

Supplement: S10 Fig — Bottom: The number of detected sequences and the minimum firing rate over all detected sequences as a function of the duration of the pre-recording in which the sequences were detected. Note that the x-axis uses a non-linear scale. Middle: For each pre-recording duration plotted in the bottom (shared x-axis), the distribution of the recall scores of the detected units when tested on a separate 2-minute part of the recording, replayed as to mimic a real time sorting. The red line reflects the average recall over all sequences detected in the pre-recording. Top: Same as middle but for precision instead of recall. Overall, the number of detected sequences increases with longer pre-recording durations. This is due to more low firing rate units being detected. Some sequences with below 1Hz firing rates can already be detected in pre-recordings of 40s. Additional units are being detected with longer pre-recording durations and this plateaus around pre-recordings of 5min. Note that the average precision and recall over all detected sequences remains consistent, independent of the pre-recording duration. (TIF) [file pone.0312438.s010.tif]

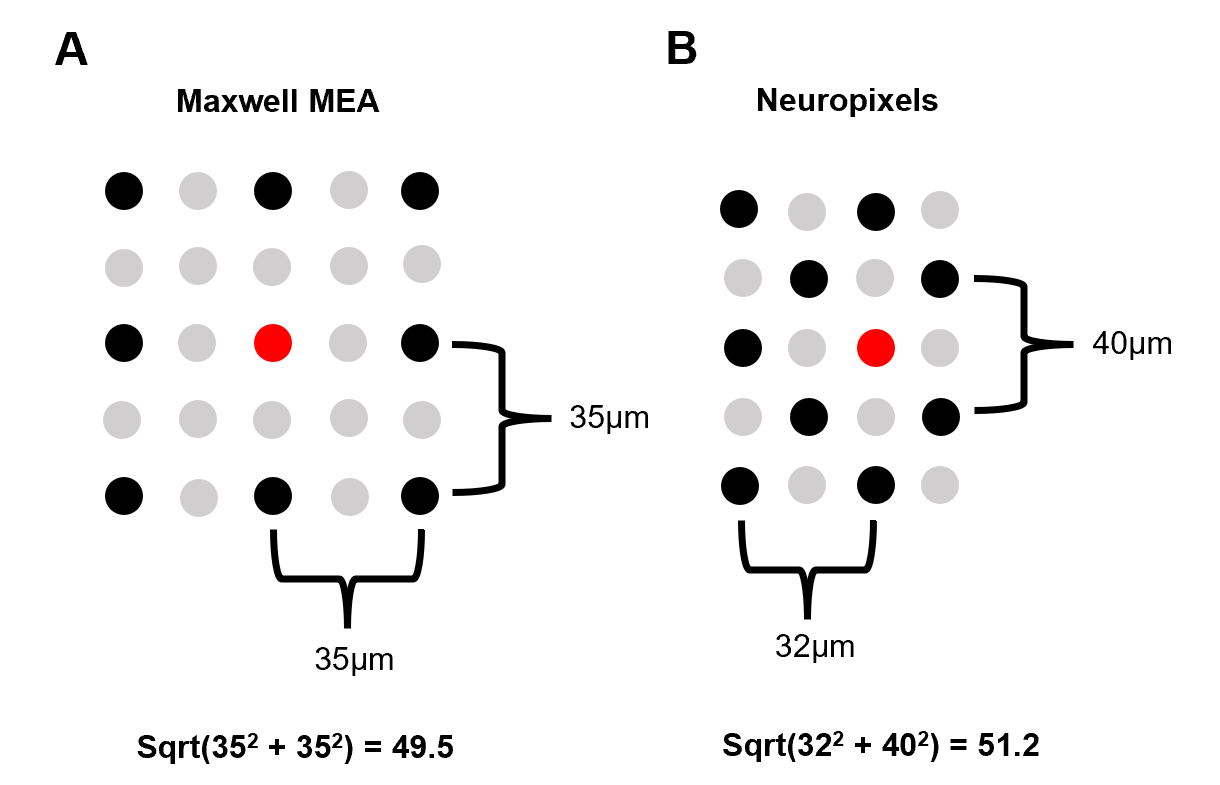

Supplement: S11 Fig — A) Schematic representation of electrode locations on an MEA where every other electrode is selected in the configuration (resulting in 35μm pitch, grey electrodes are not included in the configuration). Within a 50μm radius of the electrode in red, the closest electrodes directly above and besides the red electrode are selected, as well as the closest electrodes diagonally spaced relative to the red electrode. B) Schematic representation of electrode locations on a Neuropixels probe with checkerboard configuration (grey electrodes are not included in the configuration). Within a 50μm radius of the electrode in red, the closest electrodes above and besides the red electrode are selected, as well as the closest electrodes diagonally spaced relative to the red electrode. (TIF) [file pone.0312438.s011.tif]

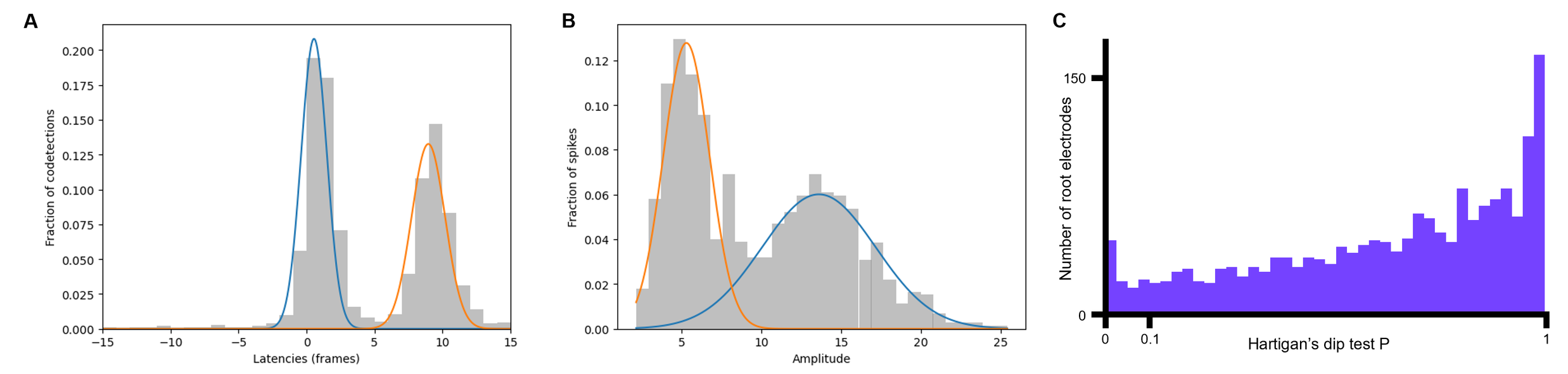

Supplement: S12 Fig — A) Example of an interelectrode interval distribution for all splitting codetections of a contaminated loose electrode on the Neuropixels recording of Fig 4. The bimodal distribution reflects a propagation from two different axons. The decision boundary of the 2-component Gaussian mixture model splits the interelectrode intervals into the two separate preliminary propagations sequences. The blue and orange curves show the probability density function of the two clusters. B) Example of an amplitude distribution for all amplitudes on the root electrode of a contaminated preliminary propagation sequence on the Neuropixels recording of Fig 4. The bimodal distribution (Hartigan’s dip test, P = 0.0005) reflects a propagation from two different axons. The decision boundary of the 2-component Gaussian mixture model splits the interelectrode intervals into the two separate preliminary propagations sequences. The blue and orange curves show the probability density function of the two clusters. C) Distribution of the Hartigan’s dip test P-values used for splitting the preliminary propagation sequences based on the amplitude of the root electrode in the Neuropixels recording of Fig 4. A distribution is considered multimodal if P<0.1. (TIF) [file pone.0312438.s012.tif]
